# Mirror-Like Mechanisms and Music

**DOI:** 10.1100/tsw.2009.160

**Published:** 2009-12-16

**Authors:** Alessandro D'Ausilio

**Affiliations:** DSBTA - Section of Human Physiology, University of Ferrara, Italy

**Keywords:** mirror neurons, neuroscience of music, audio-motor integration, motor system, premotor cortex

## Abstract

The neural processes underlying sensory-motor integration have always attracted strong interest. The classic view is that action and perception are two extremes of mental operations. In the past 2 decades, though, a large number of discoveries have indeed refuted such an interpretation in favor of a more integrated view. Specifically, the discovery of mirror neurons in monkey premotor cortex is a rather strong demonstration that sensory and motor processes share the same neural substrates. In fact, these cells show complex sensory-motor properties, such that observed, heard, or executed goal-directed actions could equally activate these neurons. On the other hand, the neuroscience of music has similarly emerged as an active and productive field of research. In fact, music-related behaviors are a useful model of action-perception mechanisms and how they develop through training. More recently, these two lines of research have begun to intersect into a novel branch of research. As a consequence, it has been proposed recently that mirror-like mechanisms might be at the basis of human music perception-production abilities. The scope of the present short review is to set the scientific background for mirror-like mechanisms in music by examining recent published data.

## MIRROR AND CANONICAL NEURONS IN MONKEY F5

The motor system has been classically considered as an output module for planning and executing motor commands[1], organized into a hierarchical structure that contains abstract motor intentions down to motor implementation parameters in dissociable cortical, subcortical, and spinal regions[2]. For instance, on the abstract end of this continuum, neurophysiological research has shown that in monkey area F5, a premotor area, complex hand and mouth movements are represented[3] (see Fig. 1A). Neurons in this region discharge during the execution of goal-directed actions (i.e., grasping, manipulating, tearing, or holding), whereas they do not discharge during similar movements made with other purposes. More interestingly, they become active during movements that have an identical goal regardless of the effectors used for attaining it. This evidence suggests that F5 premotor neurons are able to generalize action goals. In addition to their motor properties, however, several F5 neurons also show complex visual responses (visuomotor neurons). Two categories of these visuomotor neurons are present in area F5: canonical and mirror neurons. Canonical cells discharge when the monkey observes graspable objects or executes

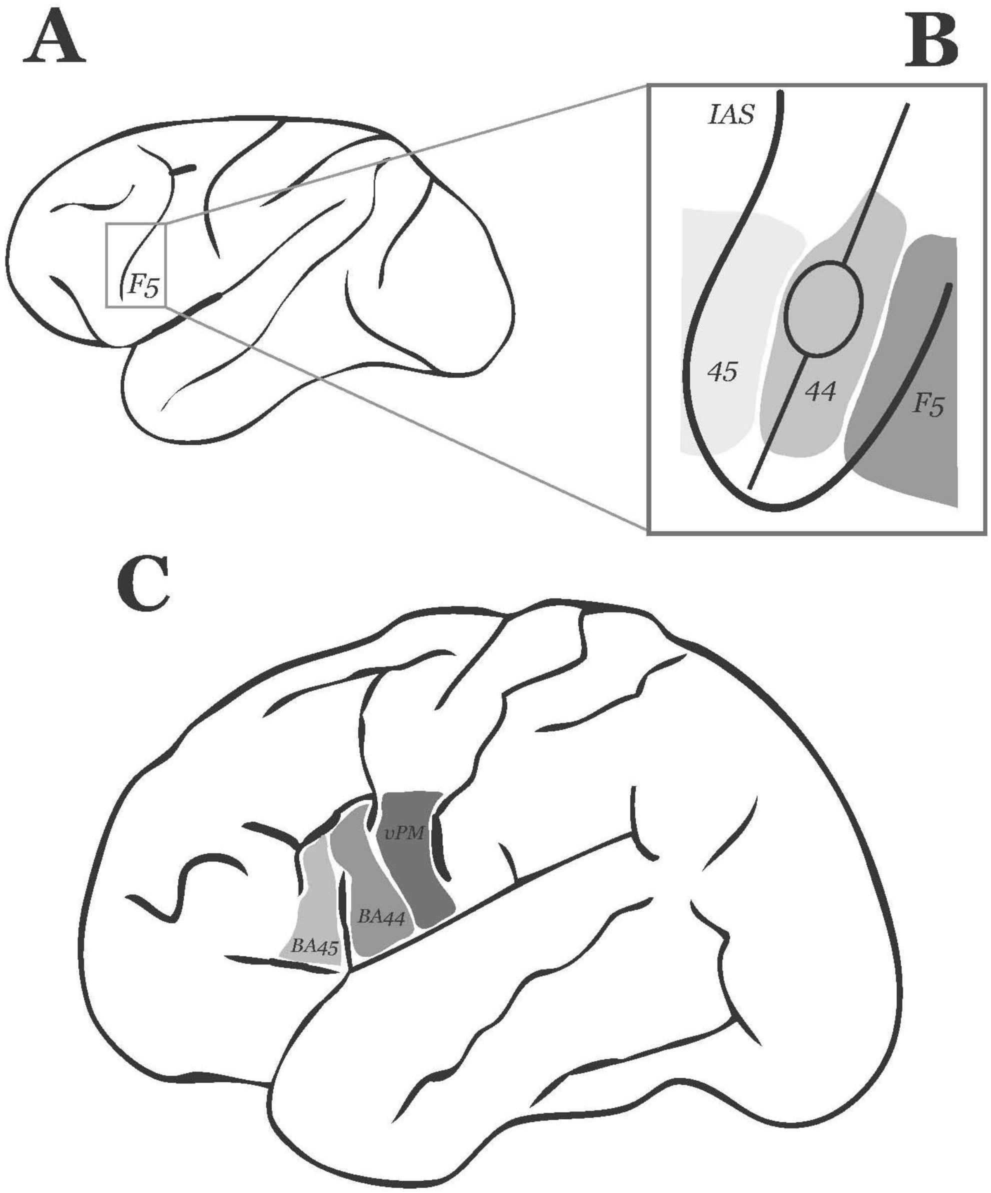

**FIGURE 1.** Panel A shows the gross anatomy of the macaque monkey brain. This sketch represents a lateral view showing, in the box, the portion of cortex where region F5 is located. Panel B shows the unfolded inferior portion of the arcuate sulcus (IAS). Monkey area 44 lies in the fundus of the sulcus just anterior to area F5 and posterior to area 45. Panel C shows a lateral view of the human brain showing the anatomical location of areas BA44, BA45, and ventral premotor cortex (vPM). These areas share important cytoarchitectonic properties with monkey areas 44, 45, and F5.

grasping actions upon those objects[4]. Mirror neurons discharge both when the monkey executes and observes another individual making the same action in front of it[5]. Visual and motor properties of canonical neurons show a strict congruence between the two types of responses (i.e., a neuron active when the monkey observes small-size objects also discharge during precision grip). The most likely interpretation for the visual discharge of canonical neurons is that there is a close link between the most common 3D stimuli and actions necessary to interact with them. Mirror neurons, instead, fire when the monkey acts on an object and when it observes another individual making a similar goal-directed action. Typically, mirror neurons show congruence between observed and executed actions.

## MIRROR AND CANONICAL MECHANISMS IN HUMANS

Recent comparative cytoarchitectonic studies of human and monkey frontal cortices looked for the human homologue of monkey area F5, where mirror and canonical neurons were originally found. Area F5 is a typical, premotor area characterized by no granular layer IV, analogous to human ventral premotor cortex (vPM). The pars opercularis (BA44, the posterior part of Broca's area) has a rudimentary layer IV and no large cell bodies in layer III. Human BA44, a disgranular cortex, shows important similarities with a monkey brain area in the fundus of the inferior branch of the arcuate sulcus. The pars triangularis (BA45, the anterior part of Broca's area), instead, is characterized by an important granular layer IV and very large cell bodies in layer III, suggesting that this area is substantially a prefrontal region[6] (see Fig. 1B,C). This structural difference between granular cortex (BA45) on one side, and agranular (vPM) and disgranular (BA44) cortices on the other, is also paralleled by recent connectivity studies in both humans[7] and monkeys[8]. These studies demonstrate that the former (BA45) is anatomically linked to temporal areas, whereas the latter two (vPM and BA44) are mostly connected to the inferior parietal lobule and the temporoparietal junction. Therefore, human BA44 and vPM are the best candidates to host neuronal populations with mirror- and canonical-like properties.

On the functional side, a growing body of neuroimaging evidence indeed indicates that vPM and posterior Broca's area (BA44) appear to have properties similar to monkey area F5. Passive observation of graspable objects, in accordance with canonical-neuron properties in the monkey, was found to elicit motor and vPM activities in humans[9]. The vPM cortex also became active during the simple observation of tools[10]. Several other experiments studied brain activity when the participants observed actions of others, in search of mirror-like functions. Activations were present in BA44 and vPM cortices with a functional pattern analogous to that of mirror neurons in the monkey[11,12]. Neuropsychological studies have similarly demonstrated that apraxic patients with lesions centered in the pars opercularis and in the pars triangularis of the left inferior frontal gyrus (IFG) have problems in gesture comprehension[13]. Moreover, frontal aphasics without apraxia failed in an action-sequencing task, further suggesting the intriguing possibility that Broca's area could represent the hierarchy of action goals, seen or executed, rather than the basic motor program to execute them[14].

## AUDITORY MIRROR MECHANISM

Beside the visuomotor properties of mirror neurons, it has been shown that a subset of those cells do respond also to the sound of actions. Some of these cells have, in fact, trimodal (motor, visual, and auditory) properties[15]. These cells (and the network they are part of) may thus encode a multimodal representation of action goals that may be retrieved in different modalities. Moreover, these data have fostered studies on the auditory component of the human mirror-like mechanism. This putative mechanism is thought to map the acoustic representation of actions into the motor plans necessary to produce those actions. Auditory mirror properties have been shown mostly using functional magnetic resonance imaging (fMRI), magnetoelectroencephalography (MEG), electroencephalography (EEG), or transcranial magnetic stimulation (TMS). For instance, a recent fMRI paper showed premotor activities when listening to affective nonverbal vocalizations. Activations were similar to those shown during the execution of facial movements[16]. On the other hand, several studies showed converging evidence that listening to human action-related sounds activates the motor system[17,18]. More interestingly, listening to action-related sounds, typically executed by the hand or the mouth, activate motor or premotor areas according to the typical dorsoventral action somatotopy[19,20]. Similarly, tool-related sounds activate the same premotor areas for tool observation and pantomime[21] only if they are associated with tools the subject can use (i.e., telephone ringing vs. piano sounds in nonmusicians[22]). It is interesting to note that, in agreement with monkey data, the auditory presentation of actions or tool use activates motor areas typically involved in action execution and observation. Finally, in agreement with the above-mentioned studies performed on healthy subjects, it has been shown that apraxic patients are impaired in their ability to understand action-related sounds[23]. Therefore, functional neuroimaging and patients' data agree on the possibility that action encoding in the motor system can be accessed via both auditory and visual display of actions.

## MUSIC AND THE MIRROR MECHANISM

The ability to play music is complex and fascinating, and requires years of practice to obtain the intricate coordination of different skills. Expert musicians are indeed an extraordinary population to study on how specific training can shape somatosensory[24], motor[25], and auditory representations[26], as well as multimodal integration networks[27]. In fact, musicians were utilized in several studies investigating long-term structural[28] and short-term functional[29] plastic changes in the brain. Sensory-motor integration in music could be the result of repeated co-occurrence of actions and the associated sounds, a mechanism that shows similarities with the oral-aural loop, but with the additional advantage of being spread with different degrees of expertise among the population[30]. Musicians, through years of practice, develop a strict association between a musical note, its visual representation, and the related movement[31].

In analogy with studies presented above, recent research has investigated the neural substrate of listening to music. These studies, using a variety of methods, have specifically looked for motor activities recruited by passive listening to music, just like action-related sounds or tool sounds. For instance, enhanced MEG activity while listening to piano pieces was found both in pianists[32] and naïve subjects[33] in frontal sites possibly associated to motor and premotor areas. Several fMRI studies have also looked for the shared brain substrate of music perception and production. These studies, although with several important differences, have all searched for common activations during overt production of movements (i.e., pantomime of keyboard playing) and passive listening. From these studies, it has emerged that listening to a musical piece, in expert musicians, activates a wide temporo-parieto-frontal network, including motor and premotor areas shared with musical execution[34,35,36,37]. A TMS study further verified whether active training on a musical piece was able to influence motor cortical facilitation induced by passive listening[38]. We reported that passive listening to the trained piece increased motor facilitation after as early as 30 min of practice, thus showing that such audio-motor channels in experts are subject to specific short-term plasticity.

In parallel, behavioral studies examined the effects of this audio-motor functional link in musicians. A specific experimental procedure has been developed in which the participant receives a visual instruction to perform an action (play a musical score on a piano) while, at the same time, an auditory stimulus (piano sound), which can be incongruent or congruent with that action, is presented. Using this procedure, Drost et al.[39] found longer reaction times in the incongruent condition than in the congruent condition. The same result was found with single notes[39] or chords[40], and was shown to occur only when auditory notes have the timbre of the musical instrument the participant is accustomed to play (e.g., guitar notes triggered by piano strokes do not elicit the effect[41]). Recently, experiments derived from those of Drost and colleagues tackled the temporal deployment of these effects by altering the stimulus onset asynchrony between audio and visual score presentation[42]. We found a strong facilitation when the sound preceded the imperative visual stimulus by 200 and 300 msec. Differently, the interference was effective in a time window closer to the onset of the visual imperative stimulus (from –200 msec anticipation to synchronous presentation). Neither interference nor facilitation occurred when the sound was presented after the onset of the visual imperative stimulus. In summary, behavioral research demonstrates that when a sound-evoked motor representation is analogous to that necessary to execute the piano movement, the presentation of sound "A" interferes with the concurrent motor preparation for producing sound "B", while facilitating the preparation of sound "A"[39,40,41]. This effect has a precise time-course that might inform about the mechanisms of audio-motor coupling in expert musicians[42]. In fact, the timing analysis allowed us to describe the processes of action interference and facilitation as pertaining to two temporally dissociable mechanisms that govern action performance when it has to be adjusted in response to external events. We speculated that a fast mechanism is able to interfere with the planned action and a second slower mechanism is also able to facilitate motor planning. However, it is also possible that the sensory-motor resonance could be qualitatively different if the observer (listener) has to imitate action, respond with a complementary action, or simply understand action[43]. In this study, we provided a first trace of the temporal dynamics underlying sensory-motor integration in audio-motor experts[42].

Another line of research has instead verified whether controlled musical training in naïve subjects was able to reproduce the pattern of results obtained with experts. For instance, an increase in EEG motor activity was shown during passive listening to trained musical excerpts after only 20 min of training[44]. Thus, it is likely that listening to musical excerpts (after proper motor training) activates motor representations required for the actual production of those melodies, in a mirror-like fashion and in naïve subjects too. In fact, musical excerpts might be considered as actions whose motor representations are preferentially triggered by auditory stimuli. A key question is whether those actions are encoded into their simple constitutive movements or more abstract action goals. The work of Lahav and colleagues[45] directly targeted this question by designing an fMRI study where naïve subjects were scanned after having trained a series of melodies. In the scanner, they were presented with those trained melodies, new melodies containing the same notes they trained, and new melodies with new notes. They show that the left posterior IFG maps the global representation of actions (trained melodies only) and vPM code for the single auditory events that constitute the piece (new melodies with trained notes).

A further issue, deserving more accurate research, is the conditions by which a musical excerpt gains audio-motor properties in naïve subjects too. Mutschler et al.[46], in an fMRI study, for instance, compared the effect of active training with passive listening to musical pieces on the subsequent activations elicited by passive listening to those pieces. Here, the authors' aims were to disentangle the distinct contribution of motor training and auditory processes alone. They showed larger activities in the sensory-motor hand area of the left anterior insula after active training only. This result, although initial, supports the possibility that active training is a prerequisite for the effective deployment of audio-motor circuits in naïve subjects.

## CONCLUSIONS

The functional properties of neurons in monkey F5 are particularly interesting since they code abstract motor features in a multimodal manner. Populations of neurons might store a sort of ''vocabulary'' of motor actions related to hand/mouth use. The visual response of both canonical and mirror neurons may target the same motor vocabulary of the monkey motor repertoire. What may differ is the way by which "motor words" are selected; canonical properties are selected by object observation, mirror properties by the observation or listening to an action on that object. Therefore, objects and actions form this purported motor "vocabulary" represented in terms of doable actions in the environment. An analogous mechanism seems to be present in humans and specifically in a frontoparietal circuit responsible for encoding motor properties of objects, tools, and actions, via visual and auditory cues. Music is a particular domain of human cognition that enables the study of the auditory counterpart of the human mirror mechanism. In fact, music-related behaviors have already proven to be a clean model to study mirror-like processes, and the effect of extensive training on the development and plasticity of such sensory-motor brain network.

Both neuroimaging and behavioral studies support the idea that explicit and active music training induces a specific sensory-motor functional connection. This connection is evidenced by (1) the emergence of motor activities while listening to rehearsed musical excerpts[38] and (2) the effects on motor performance induced by listening to plausible action effects[42]. Converging evidence shows that the simple listening to action effects may recruit the same neural substrates necessary for execution, in experts and naïve subjects after proper training[46]. However, there might exist multiple representations with different levels of abstractness. One possibility that is emerging from the literature is that Broca's area (BA44, more specifically) may code for more abstract multimodal representations of actions as evidenced by Lahav and colleagues[45], whereas motor and premotor regions may build a one-to-one map between single sensory events and related motor programs[47,48,49]. This hierarchical organization nicely matches that of the motor system for the control of hand actions[50].

However, the data presented in this Mini-Review cannot disentangle one central problem in this field. In fact, we still have no data showing whether motor activities during passive listening to music are mere corollary effects or do take an active part in the perceptual classification processes. Future research is necessary in order to clarify this point. On partially different areas of research, however, TMS has been successfully used to solve similar issues. TMS can indeed be used to alter subjects' performance in a given behavioral task, thus showing the causal contribution granted by a given area. For example, a recent study using TMS argues in favor of a causal role played by the frontal cortex in visual action classification[51]. In fact, the application of repetitive TMS over the posterior IFG (possibly including areas BA44 and BA45) impaired subjects' performance in judging the weight of an object lifted by an actor. More recently, it has been demonstrated that dual TMS pulses applied onto the motor system selectively impaired a phonological discrimination task[52]. In this study, TMS was administered to lip and tongue representations within primary motor cortex, while subjects had to discriminate lip- or tongue-produced phonemes. Results showed a double dissociation between stimulation site and place of articulation, thus arguing for a motor contribution to phonological discrimination. These results nicely complement those obtained with classical neuroimaging and neurophysiological techniques on the motor contribution to speech perception. In fact, earlier studies showed activities in motor regions associated to speech discrimination tasks, respectively with a great temporal[53] and spatial[54] accuracy. Thus, activities located in time and space by means of complementary techniques can be subsequently interfered by the application of TMS to infer causal function-location associations. A similar approach would be more than welcome to study the role played by the motor system in music-related behavior, especially in the context of mirror-like mechanisms.
